# Prognostic impact of the cross-sectional area of the erector spinae muscle in patients with pleuroparenchymal fibroelastosis

**DOI:** 10.1038/s41598-023-44138-y

**Published:** 2023-10-12

**Authors:** Shinsuke Kitahara, Mitsuhiro Abe, Chiyoko Kono, Noriko Sakuma, Daisuke Ishii, Takeshi Kawasaki, Jun Ikari, Takuji Suzuki

**Affiliations:** 1 Department of Respirology, Graduate School of Medicine, Chiba University, 1-8-1, Inohana, Chuo-ku, Chiba-shi, Chiba, 260-8670 Japan; 2 Department of Respiratory Medicine, JR Tokyo General Hospital, 2-1-3, Yoyogi, Shibuya-ku, Tokyo, 151-8258 Japan

**Keywords:** Diseases, Medical research, Risk factors

## Abstract

Pleuroparenchymal fibroelastosis (PPFE) progresses slowly but sometimes relatively quickly, leading to decreased activities of daily living (ADL) and muscle weakness. Skeletal muscle atrophy and muscle weakness in chronic obstructive pulmonary disease (COPD) patients may be caused by cachexia and are associated with reduced ADLs and increased risk of death. However, the association between skeletal muscle mass and the prognosis of PPFE patients remains unknown. We retrospectively analysed the clinical significance of the cross-sectional area of the erector spinae muscle (ESM_CSA_), a skeletal muscle index, and predictors of mortality within 3 years in PPFE 51 patients, idiopathic pulmonary fibrosis (IPF) 52 patients and COPD 62 patients. PPFE patients had significantly lower ESM_CSA_ than IPF or COPD patients, and lower ESM_CSA_ (< 22.57 cm^2^) was associated with prognosis within 3 years (log-rank test; p = 0.006), whereas lower body mass index (BMI) showed no association. Multivariate analysis showed that ESM_CSA_ was an independent predictor of mortality within 3 years in PPFE patients (hazard ratio, 0.854; 95% confidence interval: 0.737–0.990, p = 0.036). These results suggest the importance of monitoring ESM_CSA_ in PPFE patients and that assessing ESM_CSA_ in PPFE patients could be a more useful prognostic indicator than BMI.

## Introduction

Among types of idiopathic interstitial pneumonia, idiopathic pulmonary fibrosis (IPF) is the most frequent, causing chronic progressive fibrosis predominantly at the lung base^1,2^. Pleuroparenchymal fibroelastosis (PPFE) is another chronic pulmonary fibrosis proposed by Frankel et al. in 2004^3^. PPFE differs from IPF in that there is more fibrosis in the upper lobe, and the histopathology shows fibrosis in the alveolar space, aggregation of elastic fibres in the alveolar walls, and fibrous thickening of the visceral pleura. The European Respiratory Society (ERS), American Thoracic Society (ATS), Japanese Respiratory Society (JRS), and Latin American Thoracic Society (ALAT) 2013 guidelines^2^ rank idiopathic PPFE (IPPFE) as a rare type of interstitial lung disease (ILD). PPFE is generally considered to progress slowly; however, the rapid onset of dyspnoea sometimes leads to a decline in activities of daily living (ADL), making hospital visits difficult. IPPFE typically presents with changes in body composition, and patients often complain of an emaciated physique with weight loss^4^; this weight loss often includes muscle weakness. Skeletal muscle atrophy and loss of muscle mass in COPD have been associated with decreased ADL and risk of death^5^. However, only a few studies have compared skeletal muscle mass with the prognosis of PPFE, although a lower body mass index (BMI) has been implicated as a poor prognostic factor^4^. Antigravity muscles have been reported to reflect physical activity more readily than other muscle groups^6^. The cross-sectional area of the erector spinae muscle (ESM_CSA_), part of the antigravity muscles, can recently be quantitatively assessed using computed tomography (CT) and is considered a strong predictor of survival in COPD and IPF^7–9^. Therefore, we investigated the association of the quantification of ESM_CSA_ obtained by CT with PPFE.

## Results

### Characteristics

The baseline characteristics of the included patients (PPFE [n = 51], IPF [n = 52], COPD [n = 62]) are shown in Table 1. In this study, eight definite PPFE cases were diagnosed by surgical lung biopsy, and 33 PPFE cases had fibrosis in the lower lobes. PPFE included 14 patients with secondary PPFE (secondary causes comprised hypersensitivity pneumonitis [n = 4], non-tuberculous mycobacteria [n = 4], haematopoietic stem cell transplantation [n = 3], asbestos [n = 2], and ulcerative colitis [n = 1]); secondary factors were determined by pathology or clinical diagnosis. The Japanese severity classification of patients with IPF^10^, consisting of arterial partial pressure of oxygen and oxygen saturation during exercise, was Stage I/II/III/IV in 11/1/12/28 patients. The Global Initiative for Chronic Obstructive Lung Disease (GOLD) stage of patients with COPD was I/II/III/IV in 12/22/19/9 patients. Most patients with PPFE were underweight and tended to be thinner than those with IPF or COPD. Laboratory findings showed a mild increase in Krebs von den Lunge-6 (KL-6) and an increase in surfactant protein D. The pulmonary function test results, %forced vital capacity (FVC), and % diffusing capacity of the lung carbon monoxide (D_LCO_) were not reduced, but residual volume/total lung capacity (%RV/TLC) was increased.

**Table 1:** Clinical characteristics of 51 patients with PPFE, 52 patients with IPF and 62 patients with COPD. Data are presented as median [interquartile range]. Abbreviations: BMI; body mass index, COPD; chronic obstructive pulmonary disease, D_LCO_; diffuse capacity of the lung for carbon monoxide, ESM_CSA_; The cross-sectional area of the erector spinae muscle, FVC; forced vital capacity, KL-6; Krebs von den Lunge-6, IPF; idiopathic pulmonary fibrosis, IPPFE; idiopathic pleuroparenchymal fibroelastosis, SPPFE; secondary pleuroparenchymal fibroelastosis, SP-D; surfactant protein-D, RV; residual volume, TLC; total lung capacity, NA; not available.

	PPFE (n = 51)	IPF (n = 52)	COPD (n = 62)
General			
Age, years	64 [55–70]	72.5 [68.75–76]	69 [62–76]
Gender, male/female	28/23	41/11	52/10
Observation period, days	1743 [980.5–2807.5]	NA	NA
Surgical lung biopsy, yes/no	8/43	NA	NA
Lower lobe fibrosis, presence/absence	33/18	NA	NA
Smoking, pack-years	0 [0–23.8]	38.25 [19.7–50.3]	46.9 [30.8–70.5]
Height, cm	163.0 [156.0–169.5]	164.0 [158.9–167.0]	165.1 [159.1–169.9]
Weight, kg	49.9 [40.0–57.3]	57.0 [52.8–60.0]	63.2 [54.0–70.0]
BMI, kg/m^2^	18.6 [16.5–19.7]	21.7 [20.1–24.9]	23.1 [21.1–25.1]
ESM_CSA_, cm^2^	24.38 [19.62–28.75]	30.77 [24.78–34.90]	30.58 [25.82–35.99]
	IPPFE: 23.03 [19.57–28.19] (n = 37)	Stage I/II: 33.86 [28.07–37.51] (n = 12)	GOLD I/II: 32.36 [25.96–36.66] (n = 34)
	SPPFE: 25.88 [20.68–29.25] (n = 14)	Stage III/IV 30.77 [24.78–34.90] (n = 40)	GOLD III/IV: 29.31 [25.80–34.61] (n = 28)
Laboratory findings			
KL-6, U/mL	387.6 [310.0–567.3] (n = 50)	1003.5 [784.8–1440]	NA
SP-D, ng/mL	198.0 [118.3–305.8] (n = 38)	300 [206–376]	NA
Albumin, g/dL	4.2 [4.0–4.5]	4.0 [3.7–4.1]	NA
Lymphocyte count, /µL	1425 [1112–1847]	1900 [1417–2268]	NA
Pulmonary function test			
FVC, %-pred	72.2 [58.7–90.4] (n = 49)	57.4 [52.9–69.9] (n = 46)	93.5 [80.8–107.6]
D_LCO_, %	74.9 [63.2–89.9] (n = 38)	48 [34.0–61.8] (n = 42)	NA
RV/TLC	45.5 [35.8–51.3] (n = 39)	38.8 [31.8–45.4] (n = 42)	NA
RV/TLC, %-pred	139.5 [119.7–168.2] (n = 39)	103.6 [89.5–116.0] (n = 42)	NA

### Image analysis

**Figure 1:**
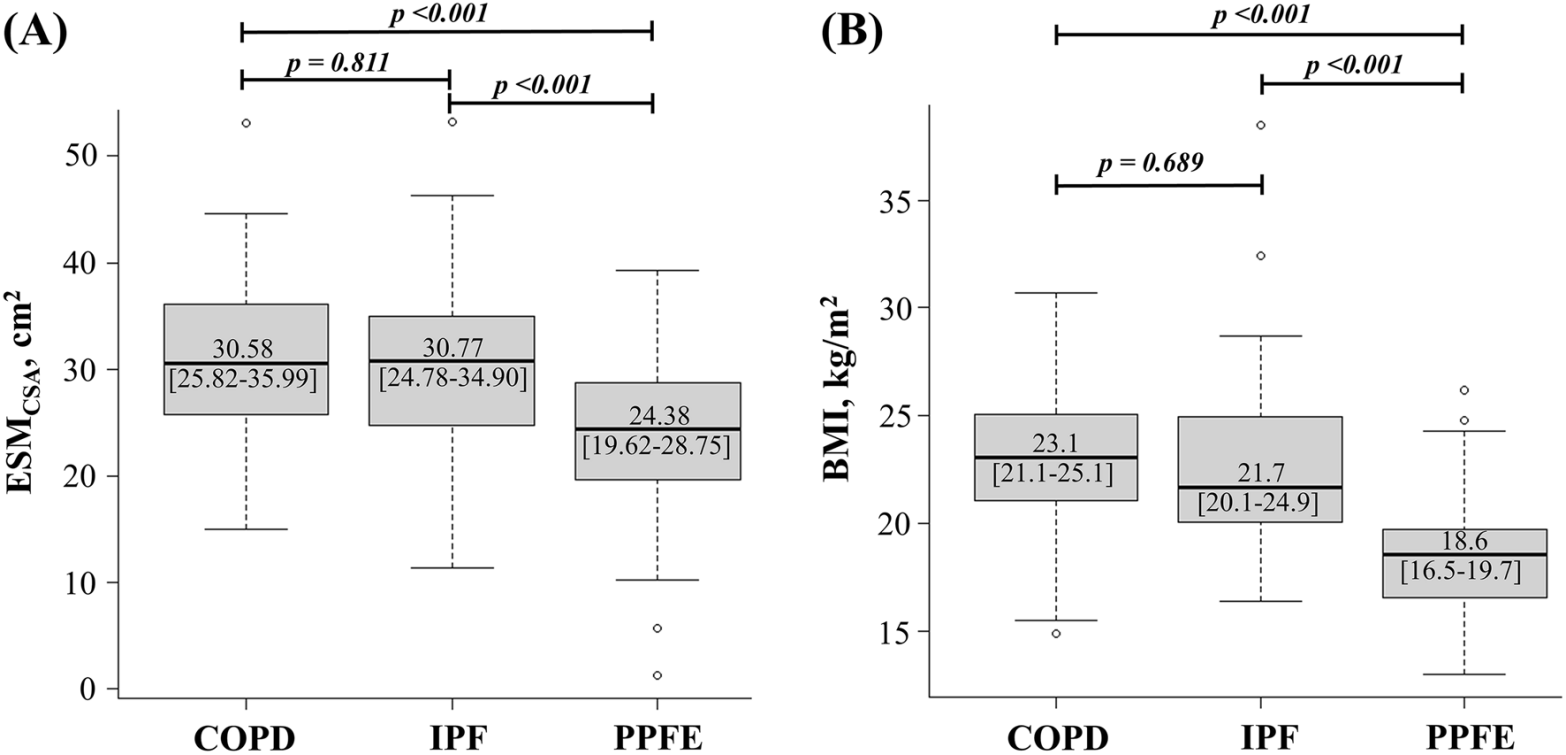
Distributions of the cross-sectional area of the erector spinae muscle (ESM_CSA_) (**A**) and body mass index (BMI) (**B**) in patients with pleuroparenchymal fibroelastosis (PPFE), idiopathic pulmonary fibrosis (IPF) and chronic obstructive pulmonary disease (COPD). Patients with PPFE had lower ESM_CSA_ and BMI than patients with IPF or COPD.

**Figure 2:**
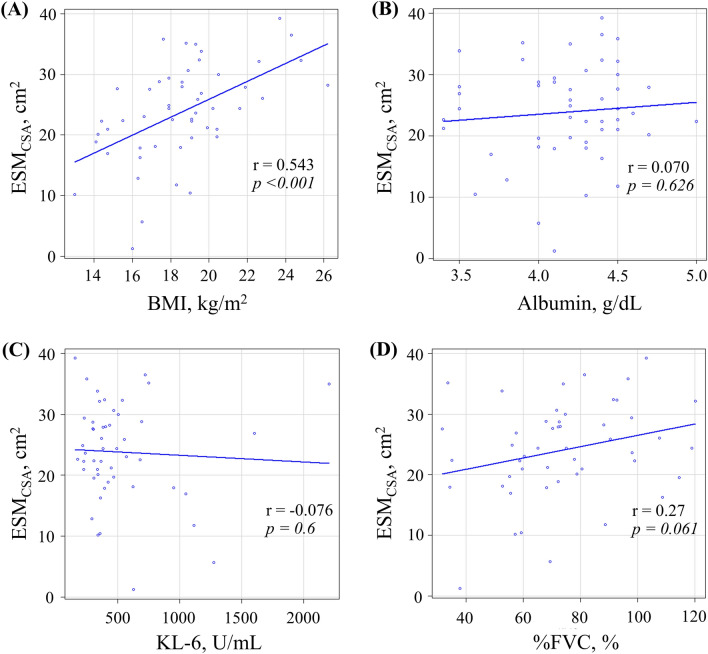
Correlations between the cross-sectional area of the erector spinae muscle (ESM_CSA_) and body mass index (BMI) (**A**), albumin (**B**), Krebs von den Lunge-6 (KL-6) (**C**) and % forced vital capacity (FVC) (**D**) in pleuroparenchymal fibroelastosis (PPFE). In patients with PPFE, there was a correlation between ESM_CSA_ and BMI but not between ESM_CSA_ and albumin, KL-6 or %FVC.

The distributions of the ESM_CSA_ and BMI are shown in Fig. 1 and Supplementary Figures 1 and 2. The ESM_CSA_ of patients with PPFE was significantly smaller than that of patients with IPF or COPD (PPFE; 24.38 [19.62–28.75] cm^2^, IPF; 30.77 [24.78–34.90] cm^2^ (Stage I/II; 33.86 [28.07–37.51] cm^2^, Stage III/IV; 30.77 [24.78–34.90] cm^2^), COPD; 30.58 [25.82–35.99] cm^2^ (GOLD I/II; 32.36 [25.96–36.66] cm^2^, GOLD III/IV; 29.31 [25.80–34.61] cm^2^); IPF vs PPFE: p < 0.001, IPF Stage I/II vs PPFE: p < 0.001, IPF Stage III/IV vs PPFE: p = 0.022, COPD vs PPFE: p < 0.001, COPD GOLD I/II vs PPFE: p < 0.001 and COPD GOLD III/IV vs PPFE: p = 0.003). BMI was significantly lower in the patients with PPFE than in those with IPF or COPD (PPFE; 18.6 [16.5–19.7] kg/m^2^, IPF; 21.7 [20.1–24.9] kg/m^2^; COPD; 23.1 [21.1–25.1] kg/m^2^; IPF vs PPFE: p < 0.001, IPF Stage I/II vs PPFE: p < 0.001, IPF Stage III/IV vs PPFE: p < 0.001; COPD vs PPFE: p < 0.001, COPD GOLD I/II vs PPFE: p < 0.001 and COPD GOLD III/IV vs PPFE: p = 0.005). The correlations among ESM_CSA_, BMI, and clinical parameters are shown in Fig. 2 and Table 2. In patients with PPFE, there was a significant correlation between ESM_CSA_ and BMI (r = 0.543, p < 0.001) or RV/TLC (r = −0.509, p < 0.001), but no correlation with KL-6 or %FVC.

**Table 2:** Correlations analyses of ESM_CSA_. Abbreviations: BMI; body mass index, D_LCO_; diffuse capacity of the lung for carbon monoxide, ESM_CSA_; The cross-sectional area of the erector spinae muscle, FVC; forced vital capacity, KL-6; Krebs von den Lunge-6, PPFE; pleuroparenchymal fibroelastosis, RV; residual volume, SP-D; surfactant protein-D, TLC; total lung capacity.

Variables	PPFE [n = 51]	
	r	p-value
Age, years	−0.337	0.002
Smoking, pack-years	0.454	< 0.001
Weight, kg	0.719	< 0.001
BMI, kg/m^2^	0.543	< 0.001
KL-6, U/mL	−0.076	0.600
SP-D, ng/mL	0.036	0.830
Albumin, g/dL	0.070	0.626
Lymphocyte count, /µL	0.207	0.144
FVC, %-pred	0.27	0.061
D_LCO_, %	0.266	0.106
RV/TLC	−0.509	< 0.001
RV/TLC, %-pred	−0.483	0.002

### Prognostic value of ESM_CSA_ and BMI in PPFE

**Figure 3:**
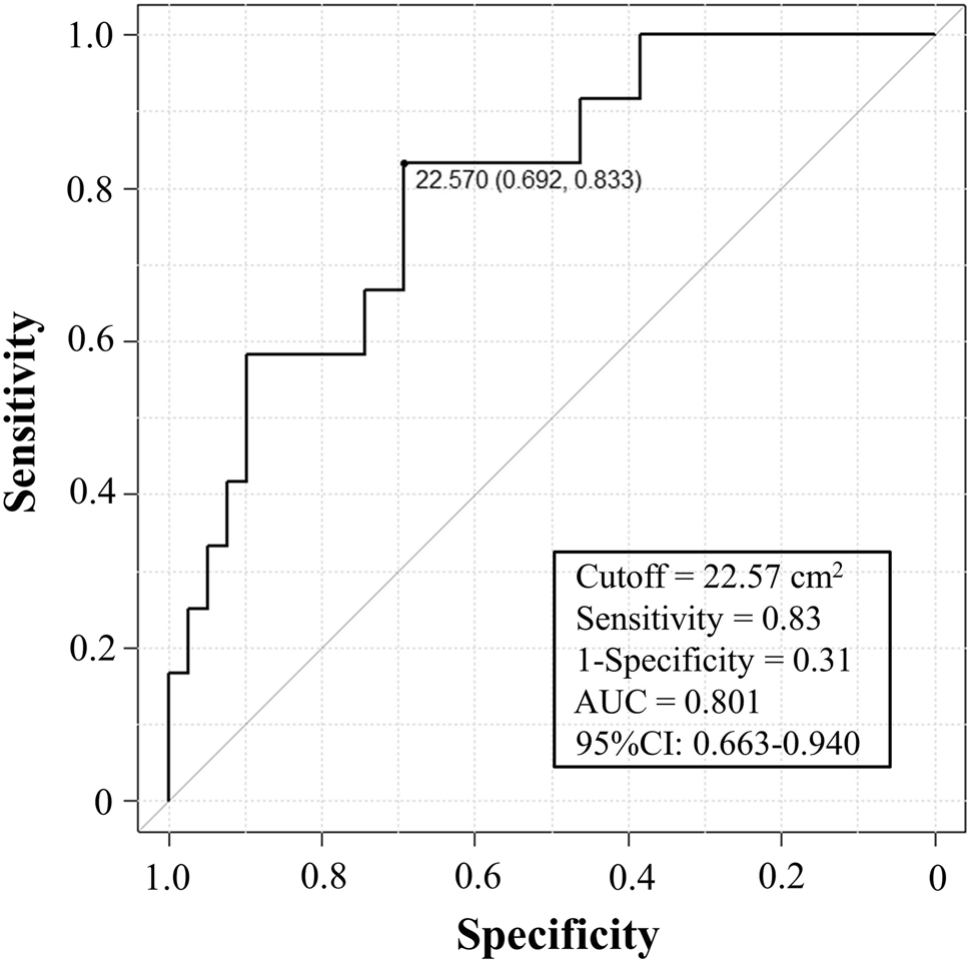
Determination of the cross-sectional area of the erector spinae muscle (ESM_CSA_) cutoff value for the risk of death within 3 years using receiver operating characteristic curves. The area under the curve was 0.801, and the ESM_CSA_ cutoff value was 22.57 cm^2^, with a sensitivity of 0.83 and a specificity of 0.31.

**Figure 4:**
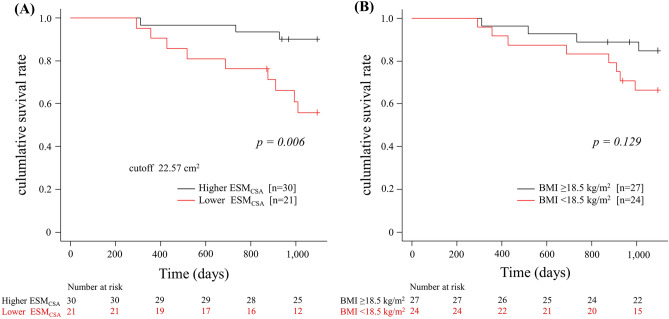
Prognostic impact of the cross-sectional area of the erector spinae muscle (ESM_CSA_) (**A**) and body mass index (BMI) (**B**) in patients with pleuroparenchymal fibroelastosis (PPFE). Low ESM_CSA_ was associated with death within 3 years in patients with PPFE. However, BMI showed no association with death within 3 years.

Over 3 years, 12 patients with PPFE died (the causes of death in patients with PPFE were respiratory failure [n = 5], acute exacerbation [n = 2], and unknown [n = 5]). The prognosis of patients with PPFE was evaluated using the Kaplan–Meier method and log-rank test, based on the ESM_CSA_ cutoff value determined by the receiver operating characteristic curve to detect the risk of death (Fig. 3) and on the BMI value (WHO definition of underweight status). The cutoff value for ESM_CSA_ was 22.57 cm^2^, with a sensitivity of 0.83, specificity of 0.31, and area under the curve of 0.801 (95% confidence interval: 0.663–0.940). In patients with PPFE, a significant difference was observed in the 3-year prognosis for ESM_CSA_ < 22.57 cm^2^ (p = 0.006) at diagnosis but not for BMI < 18.5 kg/m^2^ (p = 0.129) (Fig. 4).

### Univariate and multivariate analyses of ESM_CSA_ in PPFE

To determine the prognostic impact of measurements related to skeletal muscle changes, a preliminary Cox proportional hazards regression analysis was performed. In the univariate analysis of patients with PPFE, ESM_CSA_, age, albumin, KL-6, %FVC, and RV/TLC were associated with mortality within 3 years. In the multivariate analyses, ESM_CSA_ was independently associated with mortality within 3 years in patients with PPFE (Table 3).

**Table 3:** Prediction of 3-year mortality in patients with PPFE by univariate and multivariate Cox proportional-hazard regression analyses. Abbreviations: BMI; body mass index, D_LCO_; diffuse capacity of the lung for carbon monoxide, ESM_CSA_; The cross-sectional area of the erector spinae muscle, FVC; forced vital capacity, KL-6; Krebs von den Lunge-6, PPFE; pleuroparenchymal fibroelastosis, RV; residual volume, SP-D; surfactant protein-D, TLC; total lung capacity.

	PPFE [n = 51]	
	HR [95% CI]	p-value
Univariate analysis		
General		
Age, years	1.107 [1.026–1.194]	0.009
Gender, male	0.567 [0.180–1.787]	0.333
Lower lobe fibrosis, presence/absence	7.081[0.911–54.89]	0.061
Smoking, pack-years	1.005 [0.987–1.023]	0.590
Weight, kg	0.949 [0.896–1.006]	0.078
BMI, kg/m^2^	0.846 [0.683–1.047]	0.123
ESM_CSA_, cm^2^	0.877 [0.817–0.942]	< 0.001
Laboratory		
KL-6, U/mL	1.001 [1.000–1.002]	0.028
SP-D, ng/mL	1.000 [0.997–1.004]	0.830
Albumin, g/dL	0.114 [0.028–0.454]	0.002
Lymphocyte count, /µL	0.999 [0.998–1]	0.057
Pulmonary function test		
FVC, %-pred	0.956 [0.928–0.985]	0.003
DLCO, %	0.974 [0.946–1.002]	0.066
RV/TLC	1.109 [1.020–1.205]	0.015
RV/TLC, %-pred	1.015 [0.999–1.031]	0.059
Multivariate analysis		
Albumin, g/dL	0.028 [0.0008–1.02]	0.051
ESM_CSA_, cm^2^	0.854 [0.737–0.990]	0.036
FVC, %-pred	0.884 [0.777–1.006]	0.061

## Discussion

This study is one of the few to compare changes in skeletal muscle mass with prognosis in patients with PPFE. This study highlights the importance of these two evaluation aspects. First, a decrease in ESM_CSA_ was associated with prognosis in patients with PPFE. Second, the assessment of skeletal muscle mass in PPFE may reflect a better prognosis than BMI.

It has been suggested that a lower ESM_CSA_ may be associated with the prognosis of PPFE, including secondary PPFE (SPPFE). SPPFE is caused by haematopoietic stem cell transplantation, hypersensitivity pneumonitis, non-tuberculous mycobacterial infection, and chemotherapy^11^. However, it is possible that such comorbidities may simply be comorbid and not affect the patient and that IPPFE is included in the SPPFE. There were no significant differences in the laboratory data, respiratory complications, or survival rates between the idiopathic and secondary PPFE groups^12^. Known prognostic factors for IPPFE include older age, male, lower BMI, elevated KL-6, and lower FVC^4,13^. This study also found that older age, elevated KL-6 levels, and lower FVC were associated with prognosis; however, no previous reports have shown an association between lower ESM_CSA_ and prognosis. Suzuki et al. also reported an association between PPFE and ESM_CSA_, but no significant difference was found in prognosis. There are two possible reasons for the different results. First, the diagnostic criteria are different. Both studies had small numbers of patients and may have observed different phenotypes. Second, the disease behaviour could be different. Many of the patients in the present study were diagnosed by imaging, which means that the time axis has not been assessed. Approximately half of the patients with PPFE enrolled in this study were underweight, whereas most patients with PPFE reported by Suzuki et al. were underweight, with a BMI of < 18.5 kg/m^2^. Therefore, it is possible that the patients with PPFE in the report by Suzuki et al. had a longer disease-modifying period than those in this study and already had a more reduced skeletal muscle mass. The ESM_CSA_ in this study was independent of laboratory findings and pulmonary function tests in the multivariate analysis. Therefore, it is important to monitor ESM_CSA_ in patients with PPFE.

In this study, ESM_CSA_ was significantly correlated with age, BMI, and RV/TLC but not with %FVC or KL-6 values. The correlation of ESM_CSA_ with age and BMI could be related to a decrease in skeletal muscle mass with increasing age or decreasing body weight^14^. However, it is noteworthy that despite a significant difference in survival within 3 years for ESM_CSA_, there was no significant difference in BMI. This finding may be related to body weight components. Body weight comprises muscle mass, body fat mass, and the amount of inorganic matter in the skeleton. The lack of a significant difference in prognosis with respect to BMI may be because BMI is calculated based on body weight, including body fat and muscle mass. Weight loss can occur for various reasons, including starvation caused by reduced food intake preserving skeletal muscle mass and cachexia resulting in reduced skeletal muscle mass^15^.

Cachexia is caused by increased energy expenditure due to inflammatory-induced cytokines such as tumour necrosis factor (TNF)-α, interleukin (IL)-1 and IL-6, decreased appetite and increased protein catabolism associated with leptin and ghrelin, and skeletal muscle atrophy due to angiotensin II^16,17^. Cachexia occurs in cancer patients and is a cause of reduced skeletal muscle mass in COPD patients^18^. Compared to healthy controls, patients with COPD, IPF, and PPFE have lower ESM_CSA_^6,8^, and those with PPFE have even lower amounts of ESM_CSA_ than those with IPF^8^. In this study, the patients with PPFE had an even lower ESM_CSA_ than those with COPD or IPF. This suggests that PPFE may be more strongly affected by cachexia than COPD or IPF. Oral nutritional supplements are often used to treat cachexia, and the ghrelin receptor agonist, anamorelin, has recently proved effective in cancer patients^19,20^. Ghrelin is an appetite-enhancing peptide secreted from the stomach, which binds to the growth hormone secretagogue receptor-1a (GHSR-1a) and regulates appetite and energy metabolism^21,22^. Ghrelin and its analogues exhibit appetite-stimulating effects by promoting the expression of agouti-related neuropeptides and neuropeptide Y^23–25^ and preventing weight loss^26^. In addition to its appetite-enhancing effects, ghrelin also suppresses energy expenditure by suppressing leptin-induced inflammatory cytokines such as IL-1β, IL-6, and TNF-α, which are central to the pathogenesis of cachexia^27^, and reduces skeletal muscle catabolism induced by angiotensin II^28^. Ghrelin may also be effective for PPFE patients with possible skeletal muscle loss due to cachexia. However, non-cancer cachexia is still difficult to study compared to major diseases because of issues such as research funding and lack of public awareness. Therefore, the relationship between PPFE and cachexia, including inflammatory cytokines, remains uncertain and there are few reports on nutritional therapy and anamorelin in interstitial pneumonia, including PPFE. The symptoms of cachexia, such as severe weight loss, anorexia, early satiety, and oedema, are not clear in the early stages of the disease, and the time required for symptom onset greatly depends on the rate of progression of the underlying disease and host responses, such as activation of the systemic inflammatory response and metabolic, immune, and neuroendocrine changes^29^. It is difficult to correct undernutrition in advanced cachexia, and prevention at an early stage is considered important^30^. Although body weight and BMI are often used to assess cachexia^31,32^, the quantitative analysis of body composition using ESM_CSA_ is a better prognostic parameter than body weight or BMI^8^. The results of this study suggest that in PPFE, as in COPD, ESM_CSA_ may be a stronger prognostic factor than BMI.

This study has several limitations. First, it was a retrospective study; thus, it was impossible to assess patients' clinical symptoms, such as dyspnoea, or ADLs at the time of diagnosis of PPFE. Second, although this was not a single-centre study, the number of patients included was relatively small. Third, the study included patients with PPFE who were not pathologically diagnosed. Although pathological evaluation is necessary for a definitive diagnosis of IPPFE, surgical lung biopsy is often not performed due to the risk of postoperative lung leaks, pneumothorax, and acute exacerbation^33,34^. Therefore, Watanabe et al.^35^ proposed a method for diagnosis without surgical biopsy. Finally, the cross-sectional area of the erector spinae muscle was measured as an antigravity muscle that may influence ADL; however, other antigravity muscles, such as the iliopsoas and quadriceps muscles^36^, were not assessed. Future prospective studies are required to evaluate various skeletal muscles to overcome these limitations.

In conclusion, we investigated the relationship between ESM_CSA_ and PPFE. This study highlights the importance of monitoring ESM_CSA_ in predicting the prognosis of patients with PPFE. This suggests that ESM_CSA_ may be a better prognostic factor for PPFE than BMI.

## Methods

### Patients

This retrospective study was conducted in a cohort of 51 consecutive patients with PPFE at Chiba University Hospital and JR Tokyo General Hospital between July 2004 and June 2023. To compare skeletal muscle mass, the study enrolled 52 patients with IPF and 62 patients with COPD who visited our institute as a control group, and they were evaluated using physical measurements, pulmonary function tests, and chest CT. The control group had no malignancy, resected lungs, active infection, or neuromuscular disease.

The study protocol was approved by the Ethics Committee of Chiba University Graduate School of Medicine (M10117) and JR Tokyo General Hospital (R03-23). This retrospective study was conducted in accordance with the amended Declaration of Helsinki, and informed consent was obtained from all participants.

### Image diagnosis

The clinical diagnosis of PPFE was based on the following criteria^35^. Major criteria included (1) the presence of subpleural airspace consolidation with traction bronchiectasis of the upper lobe and (2) subpleural zonal or wedge-shaped dense fibrosis consisting of collapsed alveoli and collagen-filled alveoli with septal elastosis, with or without collagenous thickening of the visceral pleura in surgical lung biopsy specimens. Minor criteria included (1) bilateral upward migration of pulmonary hilar structures and/or volume loss in the upper lobes; (2) dry cough or exertional dyspnoea with insidious onset; and (3) RV/TLC% ≥ 115% and/or BMI ≤ 20 kg/m^2^ plus RV/TLC% ≥ 80%. The presence of major criteria 1 and 2 indicated a diagnosis of definite PPFE, and the presence of major criteria 1 and minor criteria 1, 2 and/or 3 indicated a diagnosis of clinical PPFE.

### Image analysis

Electronically stored CT images were used to assess skeletal muscle mass. All the CT images were obtained for diagnostic purposes during routine clinical practice. Chest CT was performed in the supine position with full inspiration breath-hold at 120 kV and approximately 200 mA. Scan data were analysed using the HOPE LifeMark-PACS (Fujitsu, Tokyo, Japan). Axial CT images (without contrast, 5 mm thick, 5 mm apart) taken at the inferior margin of the 12th thoracic vertebra were selected for ESM_CSA_ measurements based on previous studies. The areas of the left and right ESMs were measured by manual tracing. The sum of the left and right muscle areas was defined as ESM_CSA_. All CT analyses were independently performed by trained individuals (SK, CK, and NS) blinded to the patients' survival statuses; the results were then averaged.

### Data collection

Clinical data were obtained from the patients’ medical records. The laboratory findings and pulmonary function test results obtained at the time of diagnosis were recorded.

### Statistical analysis

Survival time was measured from the date of PPFE diagnosis and differentiated between death within 3 years and 3-year survival as early prognosis deterioration. All data are expressed as the median [interquartile range]. The Mann–Whitney U test was used to evaluate differences in means between the two groups, and the Kruskal–Wallis test and post-hoc analyses were used for multiple comparisons. The relationships between continuous variables were evaluated using Spearman's rank correlation coefficients. Univariate and multivariate analyses were performed using Cox proportional hazards regression models to determine the effect of body composition changes on prognosis. A multivariate Cox proportional hazards regression model was performed using a stepwise approach with the Akaike information criterion on explanatory variables. The ESM_CSA_ cutoff values for detecting the risk of death were determined using receiver operating characteristic curves, and BMI cutoff values were defined as underweight according to the World Health Organization. All statistical analyses were performed using R (R version 4.2.2, The R Foundation for Statistical Computing, Vienna, Austria), with p < 0.05 considered statistically significant.

### Supplementary Information

Supplementary Legends.

Supplementary Figure 1.

Supplementary Figure 2.

## Data Availability

All data generated or analysed during this study are included in this published article (and its Supplementary Information files).
