# Supplementary material for: Prognostic impact of the cross-sectional area of the erector spinae muscle in patients with pleuroparenchymal fibroelastosis
Source: Sci Rep. 2023 Oct 12;13:17289. doi: 10.1038/s41598-023-44138-y (PMC10570343; doi:10.1038/s41598-023-44138-y)
Supplement: Supplementary file 1 — Supplementary Legends. [file 41598_2023_44138_MOESM1_ESM.docx]

Supplementary Figure 1. Distributions of the cross-sectional area of the erector spinae muscle (ESM_CSA_) (A) and body mass index (BMI) (B) in patients with pleuroparenchymal fibroelastosis (PPFE) and idiopathic pulmonary fibrosis (IPF). Patients with PPFE had lower ESM_CSA_ and BMI compared to patients with IPF Stage I/II and Stage III/IV.

Supplementary Figure 2. Distributions of the cross-sectional area of the erector spinae muscle (ESM_CSA_) (A) and body mass index (BMI) (B) in patients with pleuroparenchymal fibroelastosis (PPFE) and chronic obstructive pulmonary disease (COPD). Patients with PPFE had lower ESM_CSA_ and BMI compared to patients with COPD GOLD I/II and III/IV.
