# Supplementary figures and images for: Prognostic impact of the cross-sectional area of the erector spinae muscle in patients with pleuroparenchymal fibroelastosis
Source: Sci Rep. 2023 Oct 12;13:17289. doi: 10.1038/s41598-023-44138-y (PMC10570343; doi:10.1038/s41598-023-44138-y)

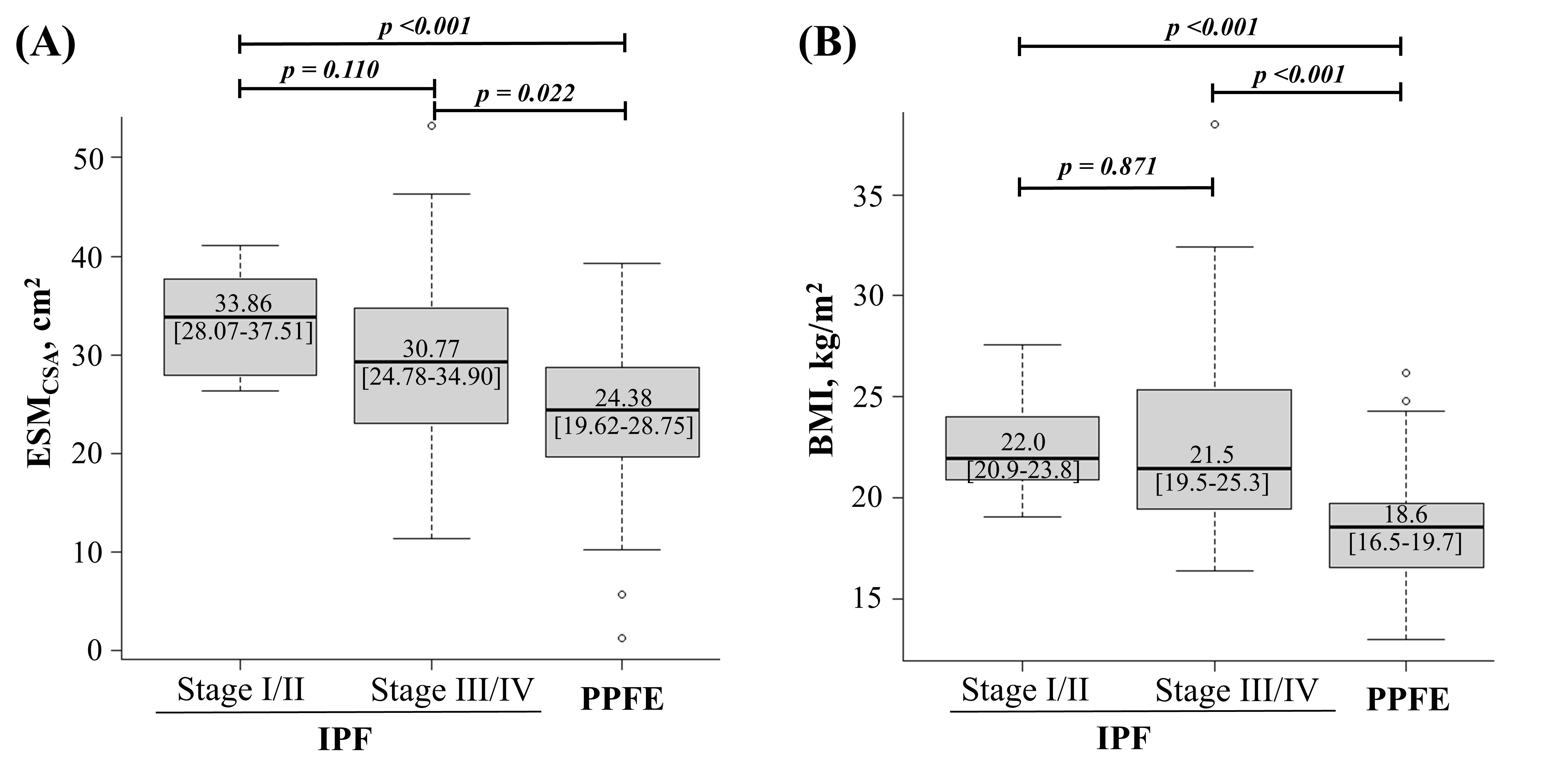

Supplement: Supplementary file 2 — Supplementary Figure 1. [file 41598_2023_44138_MOESM2_ESM.jpg]

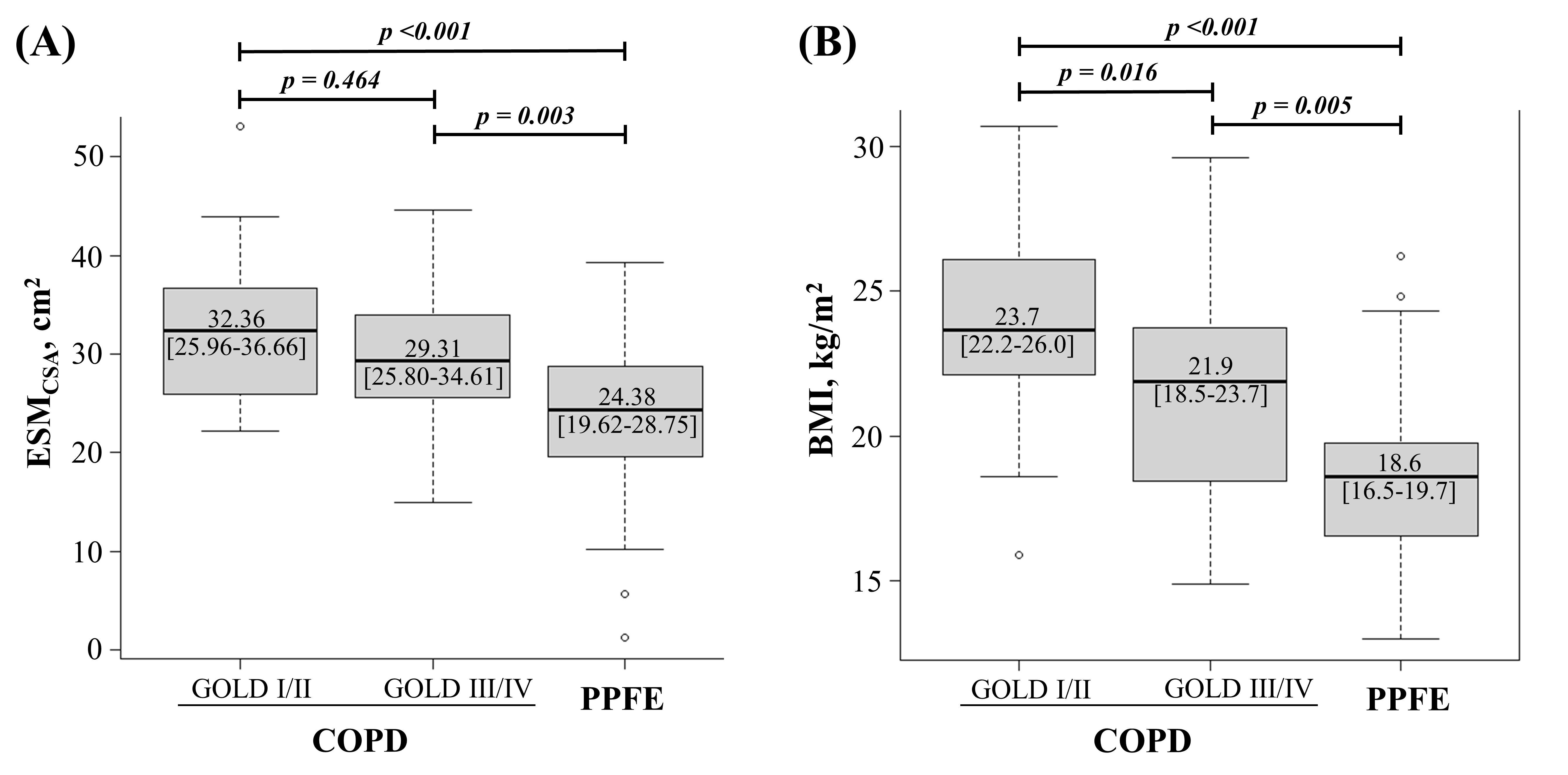

Supplement: Supplementary file 3 — Supplementary Figure 2. [file 41598_2023_44138_MOESM3_ESM.jpg]
